# Maternal Perceptions About Sensory Interventions in the Neonatal Intensive Care Unit: An Exploratory Qualitative Study

**DOI:** 10.3389/fped.2022.884329

**Published:** 2022-06-15

**Authors:** Julia Lisle, Kylie Buma, Joan Smith, Marinthea Richter, Prutha Satpute, Roberta Pineda

**Affiliations:** ^1^Chan Division of Occupational Science and Occupational Therapy, University of Southern California, Los Angeles, CA, United States; ^2^Program in Occupational Therapy, Washington University School of Medicine in St. Louis, St. Louis, MO, United States; ^3^Department of Quality, Safety and Practice Excellence St. Louis Children's Hospital, St. Louis, MO, United States; ^4^Department of Pediatrics, Keck School of Medicine, Los Angeles, CA, United States; ^5^Gehr Family Center for Health Systems Science and Innovation, University of Southern California, Los Angeles, CA, United States

**Keywords:** development, sensory, therapy, NICU (neonatal intensive care unit), qualitative study

## Abstract

**Background:**

Mothers play an important role in providing positive sensory experiences to their infants during NICU hospitalization. However, little is known regarding maternal perceptions about sensory-based interventions in the NICU. Further, understanding maternal perceptions was an important part of the process during development of the Supporting and Enhancing NICU Sensory Experiences (SENSE) program.

**Methods:**

Twenty mothers of very preterm infants were interviewed after NICU discharge and asked open-ended questions about sensory-based interventions they performed in the NICU and probed about their perceptions related to the development of a sensory-based guideline and the use of volunteers to provide sensory-based interventions when unable to be present in the NICU. Interviews were transcribed and uploaded into NVivoV.12 for content analysis.

**Results:**

Mothers reported that kangaroo care was a common sensory intervention they performed in the NICU. Of the 18 mothers who commented on the development of a sensory-based guideline, 17 (94%) said they would be accepting of one. Among 19 mothers, 18 (95%) supported volunteers conducting sensory-based interventions in their absence. Identified themes included: 1) Perceptions about development of a sensory-based guideline, 2) Perceptions of interactions with healthcare providers, 3) Maternal participation in sensory interventions, 4) Maternal experience, and 5) Emotions from mothers.

**Conclusion:**

Maternal perceptions regarding the development of a sensory-based guideline were favorable, and the SENSE program has since been finalized after incorporating important insights learned from stakeholders in this study. Mothers' perceptions were tied to their NICU experiences, which elicited strong emotions. These findings highlight important considerations when developing family-centered interventions.

## Introduction

The NICU environment is influenced by multiple factors, but the infant's sensory experiences and the role of parents in their infant's care are gaining attention as modifiable factors that could improve outcomes (1). The NICU environment can expose preterm infants to many environmental stressors, including frequent handling, persistent nociceptive pain, and sleep disruption which can adversely affect growth and development (2, 3). Further, positive forms of auditory and tactile stimulation are important for optimizing development of very preterm infants in the NICU (4) as such positive sensory exposures generate electro-cortical activity that foster an abundance of developmentally advantageous synapses (5). Parent interaction within the NICU environment is also critical, as infants have a need for human contact and nurturing (6). Parents have a long-term impact on their infant's development (7). While few studies have investigated the impact of parent presence and engagement in the NICU, those that have been conducted have identified a positive impact on infant neurobehavior by term equivalent age (8), as well as enhanced neurodevelopmental outcome in early childhood (9, 10).

However, there are barriers to optimal parent engagement in the NICU. During NICU hospitalization, parents may struggle to cope with the trauma of preterm birth, and many may experience continued stressors from the overwhelming NICU environment and witnessing their infant undergoing painful procedures (11, 12). Additionally, parents may face role alteration and feelings of inadequacy when relying on healthcare providers for assistance in performing basic caregiving tasks (11, 13, 14). Absence of succinct and non-conflicting parent education and communication can exacerbate feelings of confusion and increase their hesitance in interacting with their fragile infant (11–14). While some parents are able to overcome challenges of parenting a high-risk infant in the NICU, other parents may withdraw from the NICU knowing their infant is receiving care from the medical team (11, 12). Still others may be present at bedside, but may not have the support and/or confidence to engage with their infant (13–15).

With evidence to support the crucial role of parents, as well as the positive effects of sensory experiences for preterm infants, parents can play an important role in providing a positive sensory environment for their infant in the NICU (16, 17). However, differences in the use and interpretation of available evidence and variation in parent education and empowerment in the NICU are prevalent (18, 19). Although family-centered care is regarded as a best practice, it may not be consistently implemented (20). Nevertheless, sensory-based interventions in the NICU can be done in concert with medical interventions, and they provide promise in optimizing developmental outcomes of this fragile population (21). Due to the complex NICU environment, an active plan to engage parents and surrogate caregivers to provide sensory-based interventions to preterm infants during NICU hospitalization requires intention (22).

A guideline on evidence-based, developmentally appropriate, multi-sensory exposures for parents to conduct each day of hospitalization may decrease variability in the application of positive sensory exposures across sites, increase consistency in delivery, and empower parents to engage with their infants, while enabling more infants to gain the benefits of positive sensory experiences during NICU hospitalization. A guideline, the Supporting and Enhancing NICU Sensory Experiences (SENSE) program, was developed using a systematic and scientific process and is currently available to researchers and clinicians (23). While identifying what evidence exists on sensory exposures and obtaining NICU professional expert input were essential steps in developing the SENSE sensory-based guideline, (21, 24) gaining insights from parents of preterm infants was also vital. This study aimed to understand maternal feedback about sensory-based interventions in the NICU that was sought out during the process of SENSE program development. During the time of this study, a draft of the SENSE program had been developed, but it had not yet been finalized or trialed.

## Methods

This study was a qualitative analysis of maternal interviews and was approved by the study site Institutional Review Board. Each mother who participated gave verbal consent to participate prior to the interview.

### Participants

Twenty mothers of very preterm infants were recruited from a list of 50 participants from an overarching study which enrolled mother-infant dyads in 2015 to investigate oral feeding progression across NICU hospitalization in an 85-bed level IV NICU that is within a free-standing children's hospital and has a hybrid design (open ward and single patient rooms) (25). For the overarching study, inclusion criteria were mother-infant dyads born ≤32 weeks estimated gestational age (EGA) and hospitalized in the NICU with no congenital anomalies. Between two and 12 months following NICU discharge, mothers were contacted by phone and asked if they would participate in a 30-min phone interview to learn about their experiences with sensory-based interventions when their infant was in the NICU. Phone interviews were conducted to increase convenience for mothers with young infants at home. The range in time when mothers were contacted after NICU discharge for the interview varied due to the use of a convenience sample from the overarching study, from which infants were at various ages when the study was approved to begin. The timing of interviews was determined at the discretion and availability of the mothers. Mothers who completed the interview were given a $25 gift card.

Medical information and sociodemographic factors were collected from the medical record. In addition, a questionnaire completed by mothers prior to the infant's NICU discharge identified other social factors not available in the medical record. Factors defined included sex, EGA at birth, presence of necrotizing enterocolitis (NEC) and patent ductus arteriosus (PDA), number of days of ventilation, length of stay (LOS), maternal age at time of infant birth, race (White/Caucasian or non-White/Caucasian), maternal education (some college or no college), marital status (single or married), household income (≤ $25,000; >$25,000), distance (miles) from family home to the hospital at admission, and number of days between hospital discharge and interview. The child's corrected age (CA) at the time of the interview was also documented.

### The Interview

Semi-structured, open ended, one-on-one telephone interviews with the mothers were conducted by members of the research team who had engaged in training on interviewing and probing techniques. At the study site, mothers had experienced standard of care in the NICU, which at the time of their hospitalization, did not include the use of a sensory-based guideline. Instead, at the study site, nurses and therapists encouraged holding and skin-to-skin care based on parent and healthcare professional availability with no specific targets for dosage defined. Parents were asked about their experiences performing sensory-based interventions with their infants in the NICU, their perceptions about the development of a daily sensory-based guideline, their interactions with NICU personnel who helped them conduct sensory-based interventions, and their feelings about volunteers providing sensory interventions to their infants when they were unable to be present. While an interview script was used to gain needed information about perceptions of sensory based-guidelines, interviewers also allowed the mothers to share about other NICU experiences that the mother felt was relevant. Answers to the questions, in addition to open discussion that was led by the mother and resulted in expression of different emotions experienced during NICU hospitalization, were captured. See Appendix 1 for the interview script. Interviews were recorded, de-identified, and then transcribed.

### Data Analysis

IBM Statistical Package for the Social Sciences (SPSS 22) was used to define characteristics of the sample using descriptive statistics. The remainder of analyses were qualitative. Transcribed interviews were uploaded into NVivo V.12 for analysis. Two members of the research team used content analysis techniques (26) to independently code the mother's responses. A priori descriptive themes related to the interview guide were used to initially organize the data, and additional themes were added and collapsed as text was analyzed and grouped into nodes. Once the data was independently reviewed, coded, and organized into themes and subthemes, the two research members discussed discrepancies in coding and nodes and came to consensus. In addition, they consulted the larger research team (6 members) who came to agreement over several meetings on the final themes and subthemes represented within the data. Another author, who was not involved in the initial process of coding or discussions, then independently recoded the data for investigator triangulation. Investigator triangulation means that two or more members of the research team were involved in data analysis to bring different perspectives and observations in addition to confirmation of the findings. Definitions were clarified in a code book. A second independent researcher, who was also uninvolved in the original process, reviewed the coding, and the two independent coders came to consensus through discussion. Themes and subthemes, along with supporting narrative text, were reported. Content analysis was conducted to determine the frequency of different answers to the interview questions. This was done to further elucidate different components of the SENSE program as it was being developed.

## Results

Twenty mothers of very preterm infants participated in interviews two to 12 months after discharge from the NICU. The length of each interview varied based on information shared by each mother and ranged from 15.4 to 50.3 min. Interviews highlighted maternal perceptions about a sensory guideline, interactions with health care providers, how mothers experienced sensory interventions with their infants, their overall experience in the NICU, and the emotions felt by mothers.

See Appendix 2 for a summary of responses to the interview questions as well as quotes related to common answers to each interview question.

See Table 1 for medical and socio-demographic characteristics of the participants. Of note, two (10%) of the participants had twins and one (5%) participant had triplets.

**Table 1:** Medical and socio-demographic characteristics of participants (*n* = 20 mothers of 24 infants).

**Characteristics**	***n***	**N (%) or**
		***M (SD) or Median (IQR)***
Sex (male)	24	15 (60%)
EGA (weeks)	24	27.6 ± 2.8
NEC	22	5 (23%)
PDA	22	3 (14%)
Ventilatory support (days)	19	16 (0.00–35.00)
LOS (days)	24	93.6 ± 55.8
CA at time of interview (months)	24	7.3 ± 2.8
Maternal age (years)	24	30.6 ± 4.8
Race (White)	20	8 (40%)
Maternal education (some college)	17	11 (65%)
Marital status (single)	18	5 (28%)
Parent's income (≤ $25,000)	16	8 (50%)
Mother lives within 35 min of the hospital (driving)	17	13 (76%)
Time between hospital discharge and interview (days)	20	232.7 ± 92.3

*EGA, estimated gestational age; NEC, necrotizing enterocolitis; PDA, patent ductus arteriosus; LOS, length of stay; CA, corrected age. N is <20 when mothers did not answer questions related to that construct on the questionnaire and the information was not available in the medical record*.

See Table 2 for themes and subthemes identified. Content analysis of the transcripts identified five themes (Perceptions about Development of a Sensory-based Guideline; Perceptions of Interactions with Healthcare Providers; Maternal Participation in Sensory Interventions; Maternal Experience; Emotions from Mothers).

**Table 2:** Themes and subthemes of this study (*n* = 20).

**Themes**	**Subthemes**
Perceptions about development of a sensory-based guideline	Protocol Trainings Volunteers
Perceptions about interactions with healthcare providers	Positive (view of healthcare providers) Negative (view of healthcare providers)
Maternal participation in sensory interventions in the NICU	Barriers to participation in sensory experiences Physical environment Visitation
Maternal experiences in the NICU	Social support Roles in the NICU Transition home
Emotions from mothers	Lack of confidence Fear & Stress Frustration Pride Happiness Relief

See Tables 3–**7** for excerpts from the mothers' representative narrative text, which were used to support each subtheme.

**Table 3:** Theme 1: Perceptions about development of a sensory-based guideline.

**Subthemes**	**Narrative text/quotes**
Protocol	“I think it definitely makes a lot of sense to have a plan. Especially for the babies whose moms aren't willing to come and get in and hold their babies. (There are) also babies that aren't stimulated I just feel like, I feel like that's a great idea.” “I think that it is good to have a guide [a specific plan for sensory experiences], but being a new mom, if you gave me a time that I needed to do each thing, I think that would cause me more stress.” “I feel like a plan could definitely be beneficial, but not all babies are the same. Some babies could be hypersensitive and respond totally different than other babies…they have already been through a traumatic experience already, so they could all handle that differently.”
Trainings	“Having somebody else [nurse or therapist] there to guide you through it [participation in sensory-based intervention] was helpful. It was nice to have people there explaining it.” “I think a video would have been wonderful, that would have been great.”
Volunteers	“Other than the therapist, I feel like I would (be) happy that they [volunteers] were doing it [sensory intervention] if I wasn't able to be there, but at the same time, I would feel like I was missing out on engaging with him and helping him.” “I would be okay with all of those things [talking, playing music, massaging, holding baby, rocking baby, and giving a pacifier] in the beginning except for skin-to-skin. The more intimate the act, the harder it is.”

### Perceptions About Development of a Sensory-Based Guideline

Out of the 18 mothers who answered the question related to their perceptions about the development of a sensory guideline, 17 (94%) said they would be accepting of a sensory-based guideline, and one (6%) was not in favor. Out of the 17 mothers in favor of a guideline, thirteen (72%) mothers supported the guideline without hesitation, two (11%) had some hesitancy related to concerns about the interventions needing to be individualized, and another two (11%) had concerns about feeling overwhelmed by a guideline. Among 19 mothers who commented about volunteer involvement in the mothers' absence, 18 (95%) mothers said they would be open to having volunteers provide sensory interventions, and one mother did not support volunteer involvement (5%). Out of the 18 mothers that were open to volunteers, five (28%) said they would not want volunteers doing skin-to-skin holding. Three (15%) mothers shared interest in and preference for receiving sensory intervention training in the form of in-person education, while one (5%) expressed interest in learning by watching videos. Narrative text that supports each subtheme related to development of a sensory-based guideline can be found in Table 3.

### Perceptions About Interactions With Healthcare Providers

All 20 (100%) mothers shared stories about their interactions with healthcare providers. Five (25%) mothers described at least one negative interaction. All five of these negative experiences were attributed to poor communication and feeling that providers were rushing through interactions with the mother. Sixteen (85%) mothers reported positive interactions and relationships with NICU providers. Eight (40%) of the positive interactions were attributed to the provider teaching the mother ways to interact with or care for her infant. Three (15%) mothers described the interactions as positive, because they were being listened to by health providers; one (5%) attributed the positive experience to trusting the providers. The remaining four (20%) mothers did not expand on the reasons they viewed provider interactions as positive. Narrative text/quotations related to perceptions of interactions with healthcare providers is available in Table 4.

**Table 4:** Theme 2: Perceptions about interactions with healthcare providers.

**Subthemes**	**Quotes**
Positive (view of healthcare providers)	“but I feel like the staff, from the doctors to the bedside nurses, are very good at making me feel like my opinion was important. And you know they would consult me, now they would even encourage you to go to rounds, like doctor's rounds, to have your input.” “I felt good about (name of therapist) …I trust her.” “The physical therapist and the occupational therapist were both so helpful and they told us all the ways that we can massage, stretch, and comfort him when he is upset. Two of the nurses especially were really helpful in keeping us informed and (informed us in) ways to hold him and giving him a bath and each of the steps.”
Negative (view of healthcare providers)	“…we felt like there were some communication issues between us, the nurses, and the doctors. Possibly the things we were told by the doctors weren't the same as what we had talked to the nurses about the night before.” “But (the doctor) rushed me, you know, to go home with the NG [nasogastric] tube…But (the doctor) forced them (discharge) papers and got them [her babies] up out of there [the hospital] quick as he can and I don't know if I felt comfortable or not.”

### Maternal Participation in Sensory Interventions in the NICU

To further understand the maternal experience of implementing sensory interventions, mothers were asked to reflect upon barriers and supports to their participation in sensory-based interventions with their infant(s). Resources and education, provided by therapists, nurses, and other providers, were found to be a primary support to maternal participation in sensory interventions. Among 13 mothers who identified barriers to participation in sensory interventions, reasons included: maternal or infant sickness (*n* = 8, 62%), infant overstimulation (*n* = 4, 31%), and infant stress (*n* = 1, 8%). Among 10 mothers who reported ways that would have improved their participation in sensory interventions, mothers mentioned decreased noise (*n* = 4, 40%), lower levels of light (*n* = 1, 10%) and privacy (*n* = 5, 50%). Additionally, 13 (65%) mothers commented on the frequency of their presence in the NICU. Narrative text/quotations related to maternal participation in sensory interventions can be found in Table 5.

**Table 5:** Theme 3: Maternal Participation in Sensory Interventions in the NICU.

**Subthemes**	**Quotes**
Resources and education	“I learned so much from the NICU nurses. It was kind of terrifying, especially tracking common sense things like changing his diaper and then feeding. The therapist (taught me how to do) the massage, that was a great bonding time, and he [infant] loved it [massage], he still loves it.” “…just a book on having a preemie and what it's like in general. Not just about sensory, just in general.” “When we were there, they had a flipchart of his developmental or gestational weeks and the things that you should do, and that [format] helped a lot.”
Barriers to participation in sensory intervention	“Early on in the stay, she was still sick with the virus we all had to wear the masks and the robes so that became very cumbersome which somedays deterred us from holding her more, unfortunately.” “As much as I could, I always held him and snuggled with him and did the kangaroo care. Sometimes it was a little hard, because my C-section scars were pulling, and I had a couple of bandages so it was a little more difficult because it would irritate the scar.”
Physical environment	“You know how loud it is… the beeping, the monitors going off, the nurses talking. I don't think that (NICU) environment would be helpful when you're trying to bond and have that (sensory-based guideline)... Like those things, reading to your baby, kangaroo care, you want to be in that moment with your baby and you don't want to be distracted by other people's conversations and things.” “We would just go and get food, anywhere we could go and be alone basically because there was just no privacy in the NICU so you felt like you were on display and you're going through such a private and emotional things and there's just, everybody was watching us. But every week we would get away. Whether it was to go eat, so I could go pump. To just sit in the pump room alone, and have that quiet, that was helpful.”
Visitation	“I was there every day and did all of the sensory experiences as long as I could…I only missed a few days.”

### Maternal Experiences in the NICU

Three subthemes were identified as influencing the overall maternal experience: social support, changing roles, and transitioning home. Among 20 mothers, ten (50%) mothers reported having familial support while their infant was hospitalized in the NICU, three (15%) discussed having continued familial support during the transition home, and five (25%) mothers found other parents with infants in the NICU to be a positive social support. When considering their changing role as a mother, five (25%) of mothers explained that at the beginning of their infant's hospitalization, they were sharing caretaking responsibilities with the nurses, but as their infant got closer to discharge, the mother was able to play a larger role in caretaking. Additionally, some mothers commented on this changing responsibility and how increased participation in the maternal role positively impacted their transition home. Narrative text/quotations can be found in Table 6.

**Table 6:** Theme 4: Maternal Experience in the NICU.

**Subthemes**	**Quotes**
Social support	“My mom was mostly the one who helped me because she's been through the exact same thing I have. I was a preemie too.” “…(hospital program) ended up being a support group between the mothers. It was absolutely helpful for me to have that group. One of the first things you realize is that nobody else is going to understand.”
Roles in the NICU	“I was more of a caregiver at the beginning—and then more of a mother toward the end”
Transition home	“Well I definitely cried the first day that we came home, because it was just a total 360 or 180. I just didn't think that it would be as hard, but whenever I got home there was no nurses or no support staff. It was just me and (baby's name)…”

### Emotions From Mothers

Throughout the interviews, mothers expressed a multitude of emotions experienced during their infant's hospitalization. All 20 (100%) mothers recalled having feelings of fear and stress. Seven (35%) mothers reported feelings of decreased confidence and increased apprehension at the beginning of the hospital stay, most commonly due to the infant's size (*n* = 4, 20%), managing medical lines (*n* = 3, 15%), and limited knowledge of how to interact with their infant (*n* = 4, 20%). Additionally, holding the infant for the first time was found to be the sensory experience that evoked a broad range of emotions. Specifically, when holding their infant for the first time, six (30%) mothers stated they were scared, four (20%) stated they felt intense (yet, unspecified) emotion, and four (20%) stated they were happy. Emotions such as relief, joy, and pride were also noted throughout the interviews. The narrative text/quotations can be found in Table 7.

**Table 7:** Theme 5: Emotions from mothers.

**Subthemes**	**Quotes**
Lack of confidence	“In the beginning, I was very worried. I thought he was fragile. He was going to break. I didn't really feel comfortable holding him, and I didn't really like changing his diaper, because he was so tiny. By the end of the thing [NICU stay], I was doing it all. It didn't bother me anymore, because I knew he was going to be okay.”
Fear & stress	“After he was born, I didn't see him until three days after he was born. He was tiny. I was so scared. It was definitely different than seeing a regular baby. I didn't know if I was supposed to touch him or not.” “…he wasn't doing so well, he was septic at day 7, you know that was so stressful seeing him so sick and lethargic, from the beginning he was always kind of lethargic. At week 7, he ended up getting an E. coli infection and he was really sick and that was really stressful. And being there for the eye tests, that was crazy, so then after that on Mondays I wouldn't come that early because that was a horrible thing to watch. But those were my stressful things.”
Frustration	“Well he had ROP [retinopathy of prematurity], and they didn't tell me about it until I called a few days later and they were like “yeah we found out three days ago that he had it”, and I was like “well someone was supposed to call me” and they said “oh, no one called you? Someone was supposed to call you.”
Pride	“I was like jumping off the walls I was so happy every time he would like hiccup or every time he would make a little noise or move or something like that, I would get so excited, because you know it was so much more than what he had done before.”
Happiness	“It felt so good to have her on my chest, and to feel her next to me, it…felt so good and felt like it was right.”
Relief	“It was nice just to have her home because I felt like we were all a family again. We were, my other two, were able to play with her and bond with her. Reduced stress level from not having to travel to Children's [hospital] every day.”

## Discussion

Interviews documented valuable insights into maternal perceptions about a parent-implemented sensory-based guideline, along with additional insights into the maternal experience in the NICU and the development of the maternal role within the NICU. The key findings of this study were that this sample of mothers largely reported acceptance of the development of a sensory-based guideline. Most mothers would theoretically accept volunteers doing some sensory interventions with their infants when mothers are unable to be present in the NICU. Mothers discussed how decreased privacy, the chaotic NICU environment, and their infant's medical status impacted their own participation in sensory interventions. Mothers verbalized interest in education and training on how to engage with their infants. Mothers also reported the positive impact of communication on their relationships with healthcare providers and shared experiences that evoked a multitude of emotions during the development of their maternal role in the NICU.

The majority of mothers reported that a guideline outlining specific target amounts of daily positive sensory exposures would be helpful for parents. Mothers shared that they would prefer to learn how to participate in sensory experiences outlined by the guideline via hands-on training and checklists. A study done in 2017 found that most mothers utilized smartphones and the internet to gain NICU information (27). Incorporating the technology that mothers are already using in parent education practices may increase maternal involvement and knowledge in sensory interventions. Two mothers expressed concerns about a guideline feeling overwhelming and feared that they could not meet all of the daily requirements. However, having adequately trained volunteers supplement sensory interventions, when the mothers could not complete them, was widely supported. Mothers responded that sensory intervention techniques such as holding, rocking, talking to their baby, playing music, and massage would be acceptable for a volunteer to do, but skin-to-skin holding would not be appropriate for a volunteer, as it is an intimate bonding experience. Even though volunteers may not be able to do skin-to-skin holding, there are many sensory interventions they could participate in, which could relieve some of the stress mothers may feel when participating in a program with a daily target. Valuable insights into maternal perceptions of volunteer involvement in sensory interventions in the NICU were gained, which, to our knowledge, have not previously been reported.

Healthcare providers are an important part of NICU hospitalization. The interview responses highlighted how greatly mothers' interactions with nurses, doctors, and therapists impact their perceptions of their time in the NICU. Mothers reported having a multitude of positive and negative viewpoints toward healthcare providers. When nurses, doctors, and therapists supported participation through education and took the time to listen to mothers, mothers reported a positive attitude toward these healthcare professionals. They expressed that these interactions felt more collaborative and that their input was valued. When communication between healthcare providers and mothers was hurried or not effective, negative feelings toward providers were elicited. Mothers felt frustrated when providers gave them conflicting advice and when decisions were made on their behalf. Other studies have evaluated the dynamic between healthcare professionals and parents within the NICU and its effect on maternal and infant stay (11–14, 28). Our findings are consistent with these other studies that have found that consistent, accurate, and honest communication from healthcare providers helped parents to feel engaged in their baby's treatment with increased maternal autonomy, whereas poor communication led to feelings of isolation, exclusion, and frustration (11–14, 28). Nurses, doctors, and therapists have the capability to contribute to a positive or negative socio-emotional environment for the mother-infant dyad during a stay in the NICU.

Mothers provided their perceptions of when sensory experiences should not be used with their infant in the NICU. The most common reason was due to infant illness. Research indicates that mothers' perceptions of their infant's illness differ from healthcare professionals, with mothers generally rating their babies as sicker and having more serious illnesses than neonatologists (28). Furthermore, research has found that painful procedures done to their infants can be one of the most distressing parts of the NICU stay for parents (12), but providing non-pharmacological interventions such as breastfeeding and kangaroo care can help both the neonate and the mother (29). When discussing barriers to participation in sensory experiences, some mothers digressed and explained how lack of privacy, medical electronic noises, harsh lights, and other babies crying hindered bonding experiences such as talking and skin-to-skin holding. While private rooms may seem like the clear solution to these concerns, private rooms can decrease the amount of positive sensory stimuli an infant receives (30), and parent isolation can be exacerbated (4). Sensory-based interventions that respect infant cues, adapt to concurrent medical needs, and consider the distracting environmental factors, can be beneficial to improving the parent's and infant's NICU experience.

Mothers highlighted the positive impact that social support from fellow NICU parents, relatives, and NICU staff had during their time in the NICU and in the transition home. Additionally, the data highlights how mothers experience the phenomenon of changes in their parental role across their infant's length of stay in the NICU. Similar findings of emotional support, facilitation of parental roles in the NICU, and confidence at discharge have been observed in other studies (11, 13, 14, 31). These studies found that confidence in parental knowledge of care practices was necessary for parental role delineation and eased the transition home with their infant (11, 13, 14, 31). Healthcare providers can improve maternal involvement and role delineation by encouraging mothers to take a more active role in caring for their infant, potentially resulting in better outcomes (32). Furthermore, a recent study demonstrated the benefits of NICU's offering parents multiple types of support in the form of peer-to-peer mentorship, psychosocial support, and a family-centered approach to care (22). Together, findings from the current study and previous studies demonstrate the possible role of a sensory support team as an additional form of support that can help to empower, educate, and facilitate mother's participation in a sensory strategy, with the aim of improving infant outcomes and maternal confidence.

Finally, mothers reported experiencing several emotions while their infants were in the NICU including confidence, exhaustion and fatigue, fear, stress, frustration, pride, happiness, and relief. These emotions often came through when telling stories about interactions with healthcare providers, family members, and changes in the infant's medical status. The emotions expressed in these interviews are consistent with other findings from reports on NICU parent interviews. In a study done in 2017, it was found that parents express both positive and negative emotions, directly related to the medical complexity of their infant (12). As the infant became more medically stable, emotions such as excitement and anticipation for discharge were expressed (12). Parents' emotions must be taken into account when providing sensory interventions to their infants, fostering a relationship of trust and understanding between the parents, infants, and healthcare providers.

### Limitations

This study aimed to understand parental perspectives on the development of a sensory based guideline for parents in the NICU. However, it was conducted ahead of the SENSE program being finalized and did not capture perceptions of their actual experience with it. Further the interview guide reflected questions about the development of the sensory-based guideline, although parents led discussion into other realms such as emotions of being a NICU parent. Although several subthemes were identified during analysis, probing questions were not necessarily posed to participants during the interviews to gain more in-depth information on aspects that were not directly related to the pre-established focus area. The content analysis of this data is therefore limited in its ability to report the number of comments in each theme, as different content was raised across the interviews, and saturation was not the goal. This study was limited by a small sample size from one NICU. The socio-demographics are reported to enable consideration of context. In addition, the sample included only mothers, and therefore paternal perspectives could not be reported. Despite training in interview and probing techniques, another limitation relates to the varied skills and confidence of interviewers. Interview responses varied in depth and length. Some interviewers asked all the specified questions and expanded on these questions, while other interviewers did not. Some mothers participated in the interview almost a year after discharge and may have been subject to recall bias. Lastly, at the time of final data analysis and publication, the researchers were no longer involved at the study site and were unable to contact the mothers for further participation in the triangulation of the results.

### Recommendations for Future Studies

Future research on parent perceptions of sensory-based interventions in the NICU should include responses from multiple hospitals. Interviewing fathers to understand their perceptions would also strengthen future study designs. It would be valuable to investigate how the rate of parental engagement influences perceptions of involvement in a structured sensory intervention program. Further research can also include understanding parents' perceptions of application of the sensory-based guideline, which has now been established, and their perceptions about use of volunteers who have conducted the sensory interventions with their infants.

## Conclusion

Studies have shown that sensory stimulation is integral to infant development (33), and mothers play an instrumental role in supporting their infant during NICU hospitalization. A crucial part of this role includes providing positive sensory interventions for infants. Engaging stakeholders, such as mothers, during the development of a guideline or intervention is critical. These maternal interviews served as important stakeholder input to aid in developing the parent-driven sensory based guideline, the SENSE program (34), which is now being implemented in hospitals across the United States and abroad.

## Data Availability Statement

The datasets presented in this article are not readily available due to patient data privacy. Requests to access the datasets should be directed to the corresponding author.

## Ethics Statement

The studies involving human participants were reviewed and approved by the Washington University Human Research Protection Office. The Ethics Committee waived the requirement of written informed consent for participation.

## Author Contributions

JL contributed to data analysis, data interpretation, drafting of the manuscript, tables and figures, and critical revision. KB contributed to the literature search, drafting of the manuscript, data analysis, interpretation, and tables. JS contributed to study conception and design, interpretation of data, and critical revision. MR contributed to data interpretation, data analysis, and critical revision. PS contributed to data interpretation and analysis. RP contributed to study conception and design, data analysis, interpretation, drafting of manuscript, acquisition of data, and general over site. All authors contributed to the article and approved the submitted version.

## Funding

This work was supported by the Gordon and Betty Moore Foundation and an institutional grant awarded by the University Research Strategic Alliance at Washington University in St. Louis. Neither had a role in the design, data collection, analysis, interpretation, and writing of the manuscript.

## Conflict of Interest

RP and JS are authors of the SENSE Program. The remaining authors declare that the research was conducted in the absence of any commercial or financial relationships that could be construed as a potential conflict of interest.

## Publisher's Note

All claims expressed in this article are solely those of the authors and do not necessarily represent those of their affiliated organizations, or those of the publisher, the editors and the reviewers. Any product that may be evaluated in this article, or claim that may be made by its manufacturer, is not guaranteed or endorsed by the publisher.
