# Supplementary material for: Maternal Perceptions About Sensory Interventions in the Neonatal Intensive Care Unit: An Exploratory Qualitative Study
Source: Front Pediatr. 2022 Jun 15;10:884329. doi: 10.3389/fped.2022.884329 (PMC9240393; doi:10.3389/fped.2022.884329)
Supplement: Supplementary file 1 [file Table_1.docx]

Appendix 1:

**Parent Interview Script for Understanding Sensory-based Interventions in NICU**

In defining the term “sensory experiences”, sensory experiences could be good or bad, but I am referring to any positive experiences that you or someone else could have done with your baby. This could be putting your baby skin-to-skin, reading to your baby, rocking your baby, massaging, providing touch, playing music, or gently stretching your baby’s arms or legs. It may also include anything to help comfort your baby during potentially painful procedures, such as needlesticks.

I would like to know about your time in the NICU, specifically the sensory experiences of your

baby. Also, I would like to know when they were not possible, and how sensory experiences for your baby might be improved.

First, please tell me about one of your strongest memories of you and your baby in the hospital.

During your baby’s stay in the NICU...

- When were you able to hold your baby for the first time?
- When did you first read or talk to your baby?
- What were some ways you interacted with your baby?
  - For example, rock your baby?
    - If so, when?
  - Provide touch or massage?
    - If so, when?
  - Stretch or move your baby?
    - If so, when?
  - Encourage your baby to look at you?
    - If so, when?
      - the first day?
        - If yes, ask if they continued.
      - weeks after birth?
      - close to last day?
- Tell me about the sensory experiences of your baby in the NICU:
  - Based on answer:
    - Were there certain people who were helpful (or not helpful) in helping you interact with your baby? Nurses? Doctors? Therapists? Other family member? Other parents of preterm infants?
    - Were your interactions different at the beginning of the hospital stay compared to the end?
    - It appears you have had good experience with ____, what about:
      - Early during stay
      - When on oxygen
      - When in an isolette. The isolette is the box that your baby slept in before being moved to a crib.
- Is there anything that could have helped you do sensory experiences with your infant?
- Sometimes parents are not able to be at the hospital with the baby. Would it be okay for a trained volunteer or other staff to provide sensory experiences to your infant?
  - Based on answer
    - Okay for others to give your baby a pacifier if crying?
    - Talk to your baby?
    - Hold your baby?
    - Give massage?
    - Rock your baby?
    - Play music?
- Are there ways that sensory experiences in the NICU could be improved?
- Are there reasons why sensory experiences might not be used?
- A plan for specific amounts of sensory experiences for preterm infants in the NICU is being developed. This will include support and training on giving specific amounts of things like 3 hours of holding, 30 minutes of hearing music or spoken words, and/or 5 minutes of massage or touch being given each day the baby is in the hospital. What do you think about having a specific plan for sensory experiences for preterm infants in the NICU?
