# Supplementary material for: Maternal Perceptions About Sensory Interventions in the Neonatal Intensive Care Unit: An Exploratory Qualitative Study
Source: Front Pediatr. 2022 Jun 15;10:884329. doi: 10.3389/fped.2022.884329 (PMC9240393; doi:10.3389/fped.2022.884329)
Supplement: Supplementary file 2 [file Table_2.docx]

Appendix 2. Summary of Answers and Majority Quotes to Interview Questions

| Questions | Summary | # of Times Mentioned | Quotes |
| --- | --- | --- | --- |
| Tell me about one of your strongest memories of you and your baby in the hospital. | Skin to skin/holding | 10 | “The strongest memory was probably the day I got to see him for the first time, because we were separated for two days. And I was able to do kangaroo care with him within minutes of seeing him and that was the best experience ever.” |
|  | Seeing baby for first time | 5 |  |
|  | Surgery/sickness | 3 |  |
|  | Breastfeeding | 2 |  |
|  | Graduation from incubator | 1 |  |
| When were you able to hold your baby for the first time?  How did you feel the first time you held your baby? | Within a week | 4 | “The first time I was able to hold him I was so happy, but he had all the equipment on him. But I got to do skin-to-skin with him and get him all snuggled in there.” |
|  | Within a month | 2 |  |
|  | Day after admitted | 2 |  |
|  |  | |  |
|  | Scared | 6 |  |
|  | Emotional | 4 |  |
|  | Happy | 4 |  |
| When did you first read or talk to your baby?  Why was reading or talking to your baby important to you? | Next day after admitted | 5 | “I read to her, sang songs, put on music for her. We did that probably that next day.” |
|  | Every day | 4 |  |
|  | Within one month | 1 |  |
|  |  |  |  |
|  | Bonding experience | 4 |  |
|  | Recognize mother’s voice | 3 |  |
| What were some ways you interacted with your baby? | Holding | 7 | “Later on, we were able to feed her and change her diaper and help with her care. They let us take her temperature, they explained what all the different sensors did so later on towards the end we were comfortable getting her up ourselves. Unhooking her oxygen to get her out and re-hooking all of her sensors. We did as much as we were able to.” |
|  | Feeding | 4 |  |
|  | Talking | 3 |  |
|  | Playing music/singing | 2 |  |
|  | Administering cares | 2 |  |
|  | Reading | 1 |  |
| Tell me about the sensory experiences of your baby in the NICU. | Kangaroo care/skin-to-skin | 12 | “My favorite had to be skin-to-skin, I never missed a day. I always wore clothes that he could easily fit down my shirt, that was the best thing that any parent could ever do.” |
|  | Breastfeeding | 2 |  |
|  | Reading | 1 |  |
| Were there certain people who helped you interact with your baby? | Nurses | 9 | “The people who came in for physical therapy and stuff. Everyone was really good, and all the nurses. They would help me understand things better, and the doctors helped me understand things better.” |
|  | Therapists | 6 |  |
|  | All staff (doctors, therapists, nurses) | 6 |  |
|  | Family | 3 |  |
|  | Lactation consultant | 1 |  |
|  | Other parents | 1 |  |
| Were your interactions different at the beginning of the hospital stay compared to the end? | More confident | 7 | “In the beginning, I was very worried. I thought he was fragile. He was going to break. I didn’t really feel comfortable holding him, and I didn't really like changing his diaper because he was so tiny. By the end of the thing, I was doing it all. It didn't bother me anymore, because I knew he was going to be okay.” |
|  | No change | 3 |  |
|  | More connection with infant | 3 |  |
|  | Relationships with healthcare providers | 3 |  |
|  | Infant tolerance of sensory experiences | 2 |  |
| Is there anything that could have helped you do sensory experiences with your infant? | Nothing came to mind | 10 | “Probably the biggest thing would be probably a little more privacy. Maybe not a private room but a space to be with him.” |
|  | More privacy | 5 |  |
|  | Personal confidence with advocacy for infant | 1 |  |
|  | Books about premature infants | 1 |  |
|  | More physical space | 1 |  |
|  | Materials and equipment provided | 1 |  |
| Would it be okay for a trained volunteer or other staff to provide sensory experiences to your infant? | Yes | 18 | “Yes, I was fine with all of that. I pretty much told them I wanted them to take care of him like I would take care of him if I was there. So, if he needed something, I didn’t want them to think they were going to overstep the boundaries and do something that I didn’t want them to do. Because regardless of how I feel about it, if he needs it, that’s what I want him to have. So, if he needed to be rocked, if he needed to be picked up or anything like that and I wasn’t there to do it--I wanted them to do it.” |
|  | No skin-to-skin holding | 5 |  |
|  | No | 1 |  |
| Are there ways that sensory experiences in the NICU could be improved? | Nothing came to mind | 5 | “I think it would be nice to have a quiet hour in the big room or even a smaller room, I know that's not always possible. Probably private time would be the big one for me.” |
|  | Decreased noise/light | 5 |  |
|  | More staff | 2 |  |
|  | Private room availability | 2 |  |
|  | Access to sensory objects | 1 |  |
|  | Use of volunteers | 1 |  |
| Are there reasons why sensory experiences might not be used? | No reason | 7 | “Take into account medical factors. There were just some days when I could tell that a baby was overstimulated…” |
|  | Infant/maternal sickness | 8 |  |
|  | Infant stress | 1 |  |
|  | Infant overstimulation | 4 |  |
| What do you think about having a specific plan for sensory experiences for preterm infants in the NICU?  Why would you like having a specific plan? | Agree- like it | 13 | “I think it definitely makes a lot of sense to have a plan. Especially for the babies whose moms aren't willing to come and get in and hold their babies. It’s also babies that aren't stimulated I just feel like, I feel like that's a great idea.” |
|  | Agree- with hesitation | 4 |  |
|  | Disagree- dislike it | 1 |  |
|  |  |  |  |
|  | Have a checklist to follow | 1 |  |
|  | Mitigate confusion | 1 |  |
